# Concurrent palliative radiation with pembrolizumab for platinum-refractory urothelial carcinoma is associated with improved overall survival

**DOI:** 10.1016/j.ctro.2022.12.001

**Published:** 2022-12-10

**Authors:** Keita Nakamori, Shogo Yamazaki, Kazumasa Komura, Wataru Fukuokaya, Takahiro Adachi, Yosuke Hirasawa, Takeshi Hashimoto, Atsuhiko Yoshizawa, Takaya Ohno, Yusuke Yano, Kazuki Nishimura, Satoshi Tokushige, Taizo Uchimoto, Shutaro Yamamoto, Kosuke Iwatani, Fumihiko Urabe, Keiichiro Mori, Takafumi Yanagisawa, Shunsuke Tsuduki, Kiyoshi Takahara, Teruo Inamoto, Jun Miki, Takahiro Kimura, Yoshio Ohno, Ryoichi Shiroki, Haruhito Azuma

**Affiliations:** a Department of Urology, Osaka Medical and Pharmaceutical University, 2-7 Daigaku-machi, Takatsuki City, Osaka 569-8686, Japan; b Departmentof Urology, The Jikei University School of Medicine, 3-25-8, Nishi-shimbashi, Minato-ku, Tokyo 105-8461, Japan; c Department of Urology, Tokyo Medical University, 6-7-1 Nishi-shinjuku, Shinjuku-ku, Tokyo 160-0023, Japan; d Department of Urology, Fujita-Health University School of Medicine, 1-98 Dengakugakubo, Kutsukake, Toyoake City, Aichi 470-1192, Japan

## Abstract

**Background and Purpose:**

Pembrolizumab has now become a standard of care in metastatic urothelial carcinoma (mUC), although the treatment effect of the drug substantially differs among individuals. Emerging evidence suggests that radiotherapy exerts a synergistic effect with PD-1 blockade. We sought to elucidate the survival outcomes in patients who underwent palliative radiation with the pembrolizumab treatment.

**Methods:**

We retrospectively investigated our multi-institutional dataset of 235 platinum-refractory mUC patients treated with pembrolizumab as second-line treatment, collected from January 2018 and October 2021. Propensity score matching was performed to reduce biases by potential confounding factors for overall survival (OS).

**Results:**

With a median follow-up of 6.8 months, the median OS from the initiation of pembrolizumab was 13 months in 235 patients. Palliative radiation was performed in 71 (30.2%) patients for whom the median radiation dose and fraction were 30 Gy and 10 fractions, respectively. Irradiated sites were bone in 24 (33.8%), lymph node in 17 (23.9%), lung in 3 (4.2%), brain in 8 (11.3%), and other sites in 19 (26.8%). OS from the initiation of pembrolizumab was significantly longer in patients who underwent concurrent palliative radiation with pembrolizumab (39 patients: median OS: 21 months) than in both patients with palliative radiation before pembrolizumab (32 patients: median OS: 9 months) (p = 0.001) and those without palliative radiation throughout the follow-up (164 patients: median OS: 13 months) (p = 0.019). After the propensity-score matching by putative confounding factors, longer OS in patients treated with concurrent palliative radiation with pembrolizumab (n = 36) was still observed compared to patients without the concurrent palliative radiation (n = 36) in the pair matched cohort (median OS of 29 and 13 months, respectively, p = 0.033).

**Conclusions:**

Our findings suggest that the concurrent administration of palliative radiation with pembrolizumab offers a favorable effect on OS in platinum-refractory mUC patients.

## Highlights

• We investigated the hypothesis that palliative radiation to patients treated with pembrolizumab exerts a favorable effect on survival in 235 platinum-refractory metastatic urothelial carcinoma patients.
• Overall survival from the initiation of pembrolizumab was significantly longer in patients who underwent concurrent palliative radiation with pembrolizumab than in both patients with palliative radiation before pembrolizumab and those without palliative radiation throughout the follow-up.
• The result using propensity score matching still exhibited a longer overall survival in patients treated with concurrent palliative radiation with pembrolizumab than those without concurrent palliative radiation.

## Introduction

Metastatic urothelial carcinoma (mUC) is an aggressive malignancy, and platinum-based chemotherapy has been widely offered as the first-line treatment. Since GC (gemcitabine and cisplatin) regimen was approved by the Food and Drug Administration (FDA) with a comparable effect for clinical survival and a lower rate of intolerable treatment-related adverse events (AE) compared to the conventional MVAC (methotrexate, vinblastine, doxorubicin, and cisplatin) regimen [1], GC regimen became a standard of care for mUC patients. Nonetheless, the survival benefit for mUC patients had been restricted due to the lack of reliable subsequent therapy after the treatment failure of the first-line chemotherapy for more than a decade. In 2017, the results from KEYNOTE–045 trial demonstrated the survival benefit of pembrolizumab, a monoclonal antibody targeting programmed cell death protein 1 (PD-1), compared to second-line chemotherapy (paclitaxel, docetaxel, and vinflunine) in patients with platinum-refractory UC [2]. Since then, pembrolizumab has been widely offered to large numbers of patients worldwide as well as in Japan [3], [4].

Emerging evidence has indicated that radiotherapy could offer immunogenic effects, such as increased major histocompatibility complex (MHC) class I molecules with released neoantigens from tumors, enhanced tumor infiltration of CD8 + cytotoxic T lymphocytes (CTLs), and PD-L1 upregulation on tumor cells by IFNγ produced by CD8 + T cells, which offers plausible premises that radiotherapy exerts the synergistic effect with PD-1 blockage [5], [6], [7]. In a mouse model, the combination treatment with radiotherapy and anti-PD-L1 inhibitor showed significant growth inhibition compared with radiotherapy alone, not only in the irradiated xenograft models but also in the contralateral non-irradiated tumors, leading to improved survival [8]. Indeed, there has been a number of clinical reports showing the augmented abscopal effect in combination with PD-1/PD-L1 inhibitors, which is characterized by the tumor regression of untreated metastatic lesions following local radiotherapy [9], [10], [11]. For the treatment of mUC patients, several clinical trials are currently ongoing to uncover the clinical benefit of the combination of radiation therapy and immune-checkpoint inhibitors (ICIs) [12]. In real-world practice, palliative radiation is administrated to metastatic sites at the physician’s discretion, considering the various symptom from the metastatic sites, general status, and patient’s will. However, there has been little study to assess the real-world survival outcomes for mUC patients who were offered palliative radiation to the metastatic site during the pembrolizumab treatment.

In this multi-institutional cohort study, we investigated the real-world outcomes of patients treated with pembrolizumab to platinum-refractory mUC and sought to elucidate the survival benefit of palliative radiation onto the pembrolizumab treatment.

## Methods

We conducted the present study using a multi-institutional dataset from Osaka Medical and Pharmaceutical University (Osaka, Japan), the Jikei University School of Medicine (Tokyo, Japan), Tokyo Medical University (Tokyo, Japan), and Fujita-Health University School of Medicine (Aichi, Japan) between January 2018 and October 2021. The project was approved by the Institutional Review Board (IRB) of the principal institution (Osaka Medical and Pharmaceutical University; approval number: RIN–750–2571) and performed according to the principles of the World Medical Association Declaration of Helsinki. Written informed consent was obtained from the patients at the enrollment of the study. All the patients enrolled in the dataset were diagnosed with mUC including upper tract UC (UTUC), following the disease progression using platinum-based chemotherapy.

Pembrolizumab has been administrated either at a dose of 200 mg every-three weeks or 400 mg every-six weeks as previously approved [2], [13]. Computed tomography (CT) scan of the chest, abdomen, and pelvis was scheduled at six weeks of pembrolizumab, followed by every 12 weeks during their follow-up. Response of pembrolizumab treatment was evaluated by using RECIST version 1.1 and iRECIST [14], [15]. Palliative radiation to the metastatic site was considered and performed according to the patient's symptoms, including severe pain and spinal cord compression. Lesions to be irradiated were imaged and identified via CT simulation, and conformal treatment plans of doses and fractions were designated by board-certified radiologists among the institutes. The primary endpoint was overall survival (OS) from the initiation of pembrolizumab to the last follow-up or death of all causes. Clinical characteristics at the initiation of pembrolizumab were as follows: age (years) (<70/≥70), sex (male/female), smoking history (no/yes), the primary site of the tumor (bladder/upper tract), histology (pure UC/others), de novo mets (no/yes), metastatic sites, Eastern Cooperative Oncology Group Performance Status (ECOG-PS) (0/≥1), hemoglobin and neutrophil–lymphocyte ratio (NLP) at the initiation of pembrolizumab, palliative radiation treatment during the pembrolizumab treatment (no/yes), and types of chemotherapy regimens. Discontinuation of pembrolizumab due to the disease progression or treatment-related adverse events (irAEs) was decided at the physician's discretion and the patient’s will.

To reduce bias by potential confounding factors that affect the treatment outcomes, propensity-score matching was utilized. The following variables that could impact the outcomes were involved in the regression model: age (<70/≥70 years), the primary site of the tumor (bladder/ upper tract), ECOG-PS (0/≥1), hemoglobin level, NLR, de novo mets, and histology (pure UC/others). A 1:1 matching without replacement between the two groups was conducted by the nearest neighbor method with a 0.25-width caliper of the standard deviation for the logit of the propensity scores. The distribution of each factor was assessed by a contingency table with a Chi-square analysis. Kolmogorov-Smirnov normality was checked to assess normal distribution in continuous variables followed by performing a student’s *t*-test, or one-way ANOVA was examined to assess the difference between the variables. For variables with non-normal distribution, Wilcoxon or Kruskal-Wallis test was performed to assess the difference. The Kaplan–Meier curves were carried out to estimate the survival ratio, and a log-rank test was performed to calculate the clinical difference between the groups. On multivariate analysis, cox proportional-hazard regression models to define covariate-adjusted hazard ratios (HR) were conducted to investigate the association of clinical variables with OS. The statistical tests were two-sided, with p < 0.05 considered to delineate statistical significance. All the analyses were carried out using JMP® 15 (SAS Institute Inc., Cary, NC, USA) and GraphPad Prism software (GraphPad Software, La Jolla, CA, USA).

## Results

The clinical characteristics of all the 235 patients are summarized in Table 1. The mean age was 71.1 years old, and males accounted for 70.6% (166 patients) in the total cohort. The median OS from the initiation of pembrolizumab treatment was 13 months. During the median follow-up of 6.8 months, 121 (51.5%) patients were deceased. All patients had one or more metastatic sites including lymph nodes at the initiation of pembrolizumab treatment. Metastatic sites were as follows: liver (46 patients, 19.6%), lung (82 patients, 34.9%), bone (52 patients, 22.1%), and lymph nodes (202 patients, 85.9%). As shown in Table 2, palliative radiation was performed in 71 (30.2%) patients for whom the median radiation dose and fraction were 30 Gy and 10 fractions, respectively. Irradiated sites were bone in 26 (36.7%), lymph node in 20 (28.2%), lung in 3 (4.2%), brain in 9 (12.7%), and other sites in 22 (31.0%). There was no record of toxicity related to palliative radiation. All the cases were treated with external beam radiotherapy (EBRT), with no case in stereotactic body radiation therapy (SBRT).

**Table 1:** Patient characteristics in 235 mUC patients.

	total cohort	w/o palliative radiation	palliative radiation before pembro	concurrent palliative radiation with pembro	
variables	(n = 235)	(n = 164)	(n = 32)	(n = 39)	p value
age at the initiation of pembrolizumab [mean ± SD] (%)	71.1 ± 7.8	70.2 ± 9.5	67.1 ± 9.4	70.4 ± 8.7	0.99
≦70	104 (44.3)	73 (44.5)	14 (43.8)	17 (43.6)	
>70	131 (55.7)	91 (55.5)	18 (56.2)	22 (56.4)	
sex (%)					
male	166 (70.6)	121 (73.8)	23 (71.9)	22 (56.4)	
female	69 (29.4)	43 (26.2)	9 (28.1)	17 (43.6)	0.10
smoking history (%)					
no	91 (38.7)	57 (34.8)	15 (46.9)	19 (48.7)	
yes	144 (61.3)	107 (65.2)	17 (53.1)	20 (51.3)	0.16
primary tumor (%)					
bladder	143 (60.9)	101 (61.6)	20 (62.5)	22 (56.4)	
upper tract	92 (39.1)	63 (38.4)	12 (37.5)	17 (43.6)	0.82
histology (%)					
pure UC	223 (94.9)	160 (97.6)	31 (96.9)	32 (82.1)	
others	12 (5.1)	4 (2.4)	1 (3.1)	7 (17.9)	<0.001
*de novo* metastatis (%)					
no	146(62.1)	105(64.0)	19(59.4)	22(56.4)	
yes	89(37.9)	59(36.0)	13(40.6)	17(43.6)	0.64
metastatic sites at the initiation of pembrolizumab (%)					
liver	46 (19.6)	29 (17.7)	11 (34.3)	6 (15.4)	0.07
lung	82 (34.9)	53 (32.3)	9 (28.1)	20 (51.3)	0.06
bone	52 (22.1)	32 (19.5)	14 (43.8)	6 (15.4)	0.006
regional lymph node	91 (38.7)	63 (38.4)	15 (46.9)	13 (33.3)	0.5
non regional lymph node	111 (47.2)	78 (47.6)	14 (43.8)	19 (48.7)	0.91
number of metastatic sites at the initiation of pembrolizumab (%)					
1	113(48.1)	82(50)	12(37.5)	19(48.7)	
2≦	122(51.9)	82(50)	20(62.5)	20(51.3)	0.43
ECOG-PS at the initiation of pembrolizumab (%)					
0	106 (45.1)	75 (45.7)	12 (37.5)	19 (48.7)	
1≦	129 (54.9)	89 (54.3)	20 (62.5)	20 (51.3)	0.61
hemoglobin at the initiation of pembrolizumab [g/dl] (%)					
normal <	26 (11.1)	19 (11.6)	1 (3.1)	6 (15.4)	
normal ≧	209 (88.9)	145 (88.4)	31 (96.9)	33 (84.6)	0.24
NLR at the initiation of pembrolizumab [mean ± SD] (%)	3.7 ± 0.38				
3.7<	116 (49.4)	82 (50.0)	22 (68.8)	12 (30.8)	
3.7≧	119 (50.6)	82 (50.0)	10 (31.2)	27 (69.2)	0.006
prior chemotherapy before pembrolizumab (%)					
GC	156 (66.4)	107(65.3)	22(68.8)	27 (69.2)	
Gcarbo	38 (16.2)	33(20.1)	4(12.5)	1 (2.6)	
GCP	15 (6.4)	12(7.3)	1(3.1)	2 (5.2)	
Others	26 (11.0)	12(7.3)	5(15.6)	9 (23.0)	0.02

SD: standard deviation, UC: urothelial carcinoma, ECOG-PS: Eastern Cooperative Oncology Group Performance Status, NLR: neutrophil–lymphocyte ratio, GC: Gemcitabine/Cisplatin, Gcarbo: gemcitabine/carboplatin, GCP: Gemcitabine/Cisplatin/Paclitaxel.

**Table 2:** Demographic in 71 mUC patients treated with palliatve radiation during follow-up.

		palliative radiation before pembro	concurrent palliative radiation with pembro
variables	(n = 71)	(n = 32)	(n = 39)
palliative radiation total dose (Gy) median [range]	30 [8–64]	37.5 [20–64]	30 [8–60]
palliative radiation fraction: median [range]	10 [1–30]	14.5 [4–29]	10 [1–30]
palliative radiation site (%)			
bone	26 (36.7)	13(32.5)	13 (32.5)
lymph nude	20 (28.2)	12 (30)	8 (20)
lung	3 (4.2)	1 (2.5)	2 (5.0)
brain	9 (12.7)	3 (7.5)	6 (15)
other sites	22 (31.0)	11 (27.5)	11 (27.5)
number of radiation site(%)			
1	64	26(81.3)	38(97.4)
2	5	4(12.5)	1(2.6)
3	2	2(6.2)	–
symptom of radiation site(%)			
without symptom	14	7(21.9)	7(17.9)
pain	12	11(34.4)	12(30.8)
paralytic	8	4(12.5)	4(10.3)
others	16	10(31.2)	16(41.0)

SD: standard deviation, UC: urothelial carcinoma.

All the cases were treated with external beam radiotherapy (EBRT), with no case in stereotactic body radiation therapy (SBRT).

**Fig. 1:**
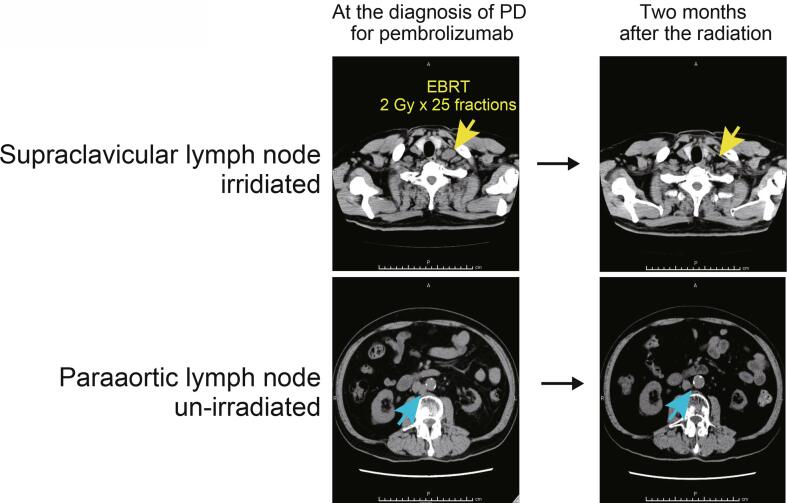
Representative case exhibiting the potential abscopal effect by concurrent palliative radiation with pembrolizumab treatment in metastatic urothelial carcinoma patients. Supraclavicular (yellow arrow) and paraaortic (blue arrow) lymph nodes progressed after second-line pembrolizumab, followed by the palliative radiation to the supraclavicular lymph node (2 Gy × 25fractions) by external beam radiation therapy (EBRT) with continuing pembrolizumab beyond disease progression. Computed tomography-two months later the radiation exhibited tumor shrinkage at not only supraclavicular but also un-irradiated paraaortic lymph nodes. (For interpretation of the references to colour in this figure legend, the reader is referred to the web version of this article.)

**Fig. 2:**
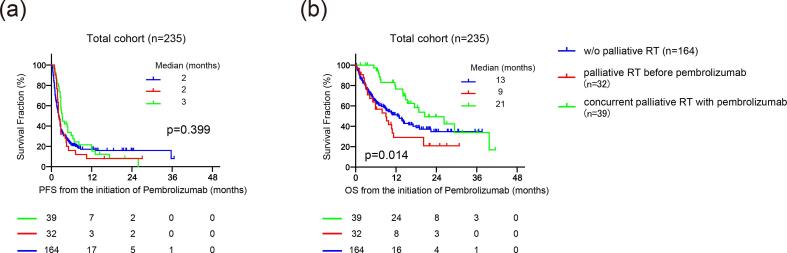
(a) Kaplan-Meier curves of progression-free survival (PFS) from the initiation of pembrolizumab for patients treated with concurrent palliative radiation with pembrolizumab (n = 39), those treated with palliative radiation before the pembrolizumab (n = 32), and those without palliative radiation throughout the follow-up (n = 164). Log-rank test was examined to determine the survival difference. (b) Kaplan-Meier curves of overall survival from the initiation of pembrolizumab for patients treated with concurrent palliative radiation with pembrolizumab (n = 39), those treated with palliative radiation before the pembrolizumab (n = 32), and those without palliative radiation throughout the follow-up (n = 164). Log-rank test was examined to determine the survival difference.

**Fig. 3:**
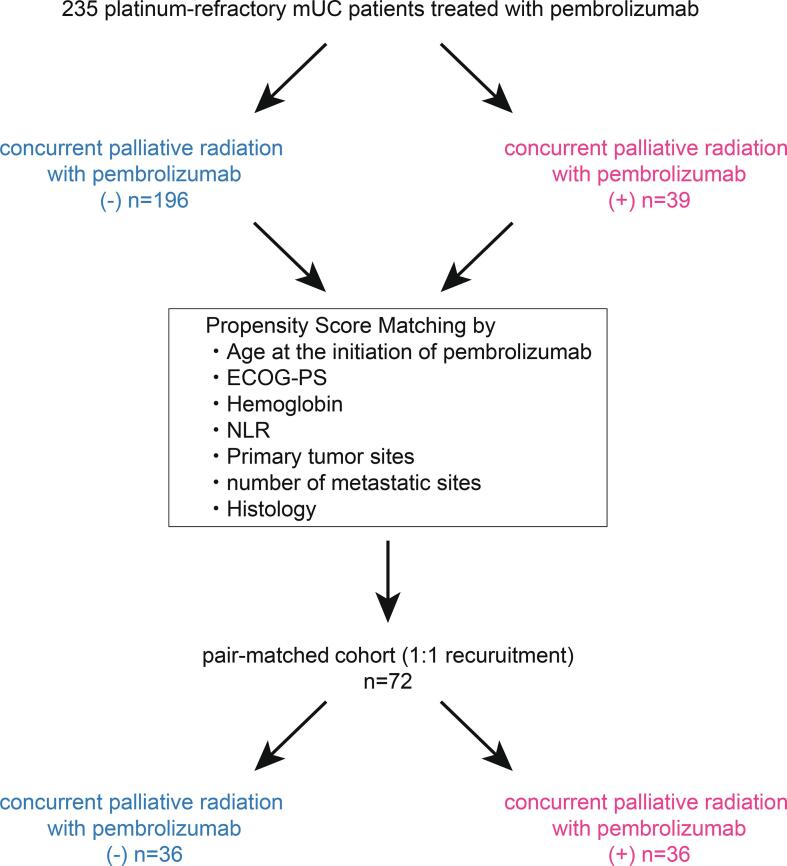
Schematic of the propensity score matching analysis to reduce bias according to the administration of concurrent palliative radiation with pembrolizumab. A 1:1 matching across the two treatment arms was conducted using the nearest neighbor method with a 0.25-width caliper of the standard deviation of the logit of the propensity scores.

**Fig. 4:**
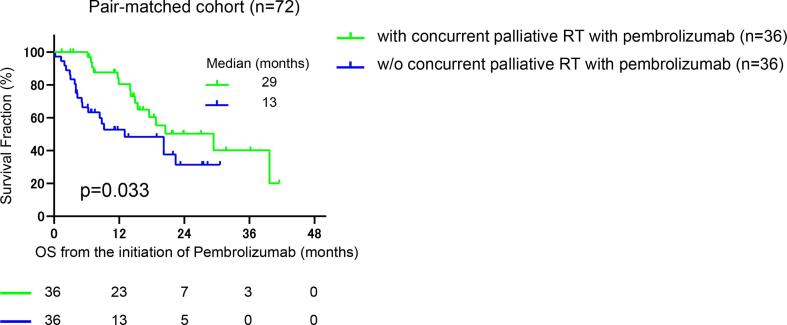
Kaplan-Meier curves of overall survival from the initiation of pembrolizumab for patients treated with or without the concurrent palliative radiation with pembrolizumab in pair-matched groups. Log-rank test was examined to determine the survival difference.

We first divided the cohort into three groups according to the administration of palliative radiation, i.e., “without palliative radiation throughout follow-up” in 164 (69.8%), “palliative radiation before pembrolizumab” in 32 (13.6%), and “concurrent palliative radiation with pembrolizumab” in 39 (16.6%) patients. The median follow-up was 6, 6, and 15 months in patients without palliative radiation (n = 164), with palliative radiation (n = 32), and with concurrent palliative radiation with pembrolizumab (n = 39), respectively. There were 96/164 (58.5%), 21/32 (65.6%), and 18/39 (46.2%) patients diagnosed with PD during their follow-up. Of them, continuing pembrolizumab beyond PD was offered in 50/164 (30.5%), 14/32 (43.8%), and 12/39 (30.7%) patients in each group, with no significant difference in the distribution among three groups (p = 0.13). Of 39 patients in the group of concurrent palliative radiation with pembrolizumab, eight patients were offered palliative radiation before the diagnosis of PD with pembrolizumab, and 31 patients underwent palliative radiation after the diagnosis of PD with pembrolizumab. Fig. 1 exhibits the representative case of the possible abscopal effect by palliative radiation treatment after PD for pembrolizumab. There was no significant difference in PFS among three groups (Fig. 2a), with median PFS of 2, 2, and 3 months in patients without palliative radiation (n = 164), palliative radiation before pembrolizumab (n = 32), and concurrent radiation with pembrolizumab (n = 39), respectively (p = 0.399). Intriguingly, OS from the initiation of pembrolizumab was significantly longer in patients who underwent concurrent palliative radiation with pembrolizumab (median OS: 21 months) than both patients with palliative radiation before pembrolizumab (median OS: 9 months) (HR: 0.38, 95%CI: 0.19–0.74, p = 0.001) and those without palliative radiation throughout the follow-up (median OS: 13 months) (HR: 0.55, 95%CI: 0.36–0.86, p = 0.019) (Fig. 2b). OS was similar between patients without the palliative radiation throughout the follow-up (median OS: 13 months) and those treated with palliative radiation before pembrolizumab (median OS: 9 months) (p = 0.231). However, we noted that the baseline characteristics among these patient groups were significantly different in several variables (Table 1). Thus, propensity score matching was utilized using putative factors including age, ECOG-PS, serum hemoglobin level, NLR, primary tumor site, number of metastatic sites, and histology (Fig. 3), from which 72 patients were deemed pair-matched groups according to the administration of concurrent palliative radiation with pembrolizumab. In the pair-matched cohort, all the variables had no significant difference between concurrent palliative radiation – and + groups (Table 3). Of note, longer OS in patients treated with concurrent palliative radiation with pembrolizumab (median OS: 29 months) was still observed compared to patients without the concurrent palliative radiation (median OS: 13 months) in the pair matched cohort (HR: 0.5, 95%CI: 0.25–0.98, p = 0.033) (Fig. 4).

**Table 3:** Clinical characteristics in propensity-score matched cohort of 72 mUC patients.

	concurrent palliative radiation with pembro		
	(-)	(+)	
variables	n = 36	n = 36	p-value
age [mean + SD] (%)	69.6 ± 9.0	70.1 ± 8.7	0.81
≦70	16(44.4)	17(47.2)	
>70	20(55.6)	19(52.8)	
sex (%)			
male	24(66.7)	22(61.1)	
female	12(33.3)	14(38.9)	0.62
smoking history (%)			
no	10(27.8)	16(44.4)	
yes	26(72.2)	20(55.6)	0.14
primary tumor (%)			
bladder	20(55.6)	20(55.6)	
upper tract	16(44.4)	16(44.4)	1.00
histology (%)			
pure UC	32(88.9)	32(88.9)	
others	4(11.1)	4(11.1)	1.00
denovo meta(%)			
no	21(58.3)	20(55.6)	
yes	15(41.7)	16(44.4)	0.81
metastatic site at the initiation of pembrolizumab (%)			
liver	6(16.7)	5(13.9)	0.74
lung	10(27.8)	17(47.2)	0.08
bone	9(25)	4(11.1)	0.12
regional lymph node	16(44.4)	11(30.6)	0.22
non regional lymph node	16(44.4)	18(50)	0.64
number of metastatic site at the initiation of pembrolizumab (%)			
1	20(55.6)	19(52.8)	
2≦	16(44.4)	17(47.2)	0.81
ECOG-PS at the initiation of pembrolizumab (%)			
0	17(47.2)	18(50)	
1≦	19(52.8)	18(50)	0.81
hemoglobin at the initiation of pembrolizumab [g/dl: mean ± SD] (%)	10.8 ± 1.6	11.0 ± 1.5	1.00
normal <	6(16.7)	6(16.7)	
normal ≧	30(83.3)	30(83.3)	
NLR at the initiation of pembrolizumab [mean ± SD] (%)	3.8 ± 3.3	4.2 ± 5.0	1.00
3.7<	10(27.8)	10(27.8)	
3.7≧	26(72.2)	26(72.2)	

mUC: metastatic urothelial carcinoma, SD: standard deviation, ECOG-PS: Eastern Cooperative Oncology Group Performance Status, NLR: neutrophil–lymphocyte ratio.

To assess the prognostic impact of the concurrent palliative radiation in mUC patients treated with pembrolizumab, we conducted the cox-regression multivariate analysis for OS from the initiation of pembrolizumab in 253 mUC patients treated with pembrolizumab (Table 4). Importantly, higher NLR (HR: 1.55, 95%CI: 1.06–2.27, p = 0.024) and the administration of concurrent palliative radiation with pembrolizumab (HR: 0.53, 95%CI: 0.32–0.89, p = 0.017) were the independent prognostic indicators of OS for platinum-refractory mUC patients treated with pembrolizumab.

**Table 4:** Cox regression multivariate analysis for OS in 253 mUC patients treated with pembrolizumab.

Variables	HR(95%CI)	P value
age (≦70/>70)	0.88 (0.60–1.28)	0.355
primry tumor (bladder/upper tract)	1.09 (0.75–1.60)	0.642
liver mets at pembrolizumab (-/+)	1.45 (0.91–2.29)	0.116
ECOG-PS (0/1≦)	1.46 (0.98–2.17)	0.062
hemoglobin (>normal/normal≧)	1.47 (0.69–3.13)	0.317
NLR (<3.7/≧3.7)	1.55 (1.06–2.27)	0.024*
concurrent palliative radiation (-/+)	0.53 (0.32–0.89)	0.017*

OS: overall survival, mUC: metastatic urothelial carcinoma, HR: hazard ratio, ECOG-PS: Eastern Cooperative Oncology Group Performance Status, NLR: neutrophil–lymphocyte ratio.

## Discussion

In the present study, we explored the possibility of survival benefits of palliative radiation treatment for platinum-refractory mUC patients treated with pembrolizumab. Our real-world outcomes exhibited that patients who underwent concurrent palliative radiation with pembrolizumab seemed to have improved OS from the initiation of pembrolizumab compared to the other patients. Since patient features were significantly different between the groups according to the timing of the palliative radiation therapy, we adjusted the effect of confounding factors among the treatment options, i.e., concurrent palliative radiation with pembrolizumab (-/+) utilizing propensity score matching analysis. This identified the pair-matched cohort of 72 patients with no significant differences among all clinical characteristics between the two groups, which allowed us to examine the difference in OS from the initiation of pembrolizumab. Our findings revealed significantly improved OS from the initiation of pembrolizumab in patients treated with concurrent palliative radiation during pembrolizumab treatment.

For mUC patients, ICIs have now become a standard of care, although the treatment effect of the drug substantially differs among patients. The results from the KEYNOTE-045 trial after more than two years of follow-up exhibited a modest progression-free survival rate (2.1 months, 95%CI: 2.0–2.2 months), ORR (21.1%, 95%CI: 16.4–26.5%), and DCR (38.5%, 95%CI: 32.7–44.6%) [16]. The two-year OS rates in their final analysis were 78.9%, 22.5%, and 9.5% with the best response for ‘CR or PR,’ ‘SD,’ and ‘PD,’ respectively. Patients with PD at their best response accounted for 48.5% with no survival benefit compared to the second-line chemotherapy. The abscopal effect is characterized by the metastatic tumor regression observed outside of the local irradiation [17]. Irradiation is known to induce immunogenic cell death characterized by releasing of tumor antigens and damage-associated molecular patterns (DAMPs) such as HSP70, HMGB1, and calreticulin [18]. These effects coordinately mediate the increased MHC class I molecules with neoantigens from dying tumor cells and cytokine stimulation, leading to the augmented tumor infiltration of CD8 + cytotoxic T lymphocytes (CTLs) [19], [20]. With the emergence of ICIs, the enhanced abscopal effect by modulating the anti-tumor microenvironment has been recognized in the real-world experience [10], [21], [22]. In 2019, Sundahl et al. demonstrated the results of a randomized phase 1 trial investigating the clinical outcomes of pembrolizumab with either sequential or concomitant stereotactic body radiotherapy (SBRT) in mUC patients. In the trial, pembrolizumab (200 mg, 3 weekly) was combined with SBRT (3 × 8 Gy, to one metastatic lesion), administered either sequentially (nine patients: prior to the first pembrolizumab treatment) or concurrently (nine patients: prior to the third pembrolizumab cycle) [23]. Intriguingly, ORR of 0% and 44% at non-irradiated metastatic lesions were observed in sequential and concomitant SBRT groups, respectively. The Median OS of each group was 4.5 months for the sequential SBRT group and 12.0 months for the concomitant SBRT group. We recently reported that the clinical effect of pembrolizumab for mUC patients previously treated with curative chemo-radiation therapy, whose tumor might have increased tumor mutation burden, has no additional survival benefit [24], [25]. Collectively, given that our findings showed an improved OS in the concurrent radiation therapy for patients treated with pembrolizumab, the enhanced effect of pembrolizumab caused by the radiation therapy should be expected only in the concurrent administration.

The current study has several limitations. The study was conducted retrospectively, and the sample size was small to conclude the results. In Japan, pembrolizumab is currently approved only in the second-line setting after disease progression of platinum-containing chemotherapy, so that we could not assess the abscopal effect for first-line ICIs. In addition, the cohort in the present study did not include patients treated with maintenance avelumab therapy following the disease control by platinum-included chemotherapies [26]. Protocols for discontinuing pembrolizumab treatment and administering palliative radiation were not standardized among the institutes. Third-line treatment including chemotherapy could impact the prognosis [27], [28]. However, we could not incorporate this concept in the present study. Due to the inconsistent protocol of immunohistochemistry among the institutes, we could not assess the clinicopathological value of PD-L1 expression. Lastly, our findings are still subject to selection bias, although we sought to address it by using a propensity score-matched model to approximate random assignment. Further studies such as prospective randomized controlled trials are warranted to prove the results of the current study.

In conclusion, we interrogated the real-world outcomes of patients treated with pembrolizumab to platinum-refractory mUC and sought to evaluate the survival benefit of palliative radiation therapy. Our data suggested that concurrent administration of palliative radiation offers a favorable effect on OS in patients treated with pembrolizumab for platinum-refractory mUC.

## Funding

This work was partially supported by the Grant-in-Aid No. 21H03070, 19K18624 (Japan Society for the Promotion of Science: JSPS), the 10.13039/100016163Takeda Science Foundation, and the Cancer Translational Research Foundation of Japanese Urological Association (JUA).

## Declaration of Competing Interest

The authors declare that they have no known competing financial interests or personal relationships that could have appeared to influence the work reported in this paper.
